# Automated Image Analysis for Detection of Coccidia in Poultry

**DOI:** 10.3390/ani14020212

**Published:** 2024-01-09

**Authors:** Isaac Kellogg, David L. Roberts, Rocio Crespo

**Affiliations:** 1 Department of Computer Science, College of Engineering, North Carolina State University, Raleigh, NC 27695, USAdlrober4@ncsu.edu (D.L.R.); 2 Department of Population Health and Pathobiology, College of Veterinary Medicine, North Carolina State University, Raleigh, NC 27606, USA

**Keywords:** Artificial Intelligence, coccidiosis, computer vision, oocyst

## Abstract

**Simple Summary:**

Coccidiosis is one of the most common and costly diseases faced by commercial poultry. To establish effective control measures, it is essential to identify the infective species and the numbers of oocysts. Standard methods for analysis require highly skilled technicians or veterinarians to manually identify and enumerate these protozoal parasites. This process is labor intensive, time-consuming, and susceptible to human error. None of the current methods available report the infectivity status of these protozoal parasites. Therefore, an automated, reproducible protocol for counting, speciation, and determination of infectivity of these protozoa using Artificial Intelligence capable of enumerating, speciating, and determining the infectivity status of the coccidia has the potential to improve diagnostics and refine control strategies to mitigate the impacts of coccidiosis on farms.

**Abstract:**

Coccidiosis, caused by the protozoan *Eimeria* sp., is one of the most common and costly diseases impacting the poultry industry. To establish effective control measures, it is essential to identify these protozoa. Typical methods for identifying and determining the severity of the protozoal infection include intestinal lesion scoring or enumeration of the protozoal oocysts in fecal samples. Standard analysis methods require highly skilled technicians or veterinarians to manually identify and manually enumerate these protozoal parasites. This process is labor intensive, time-consuming, and susceptible to human error. None of the current methods available, including molecular flow cytometry or even digital image analysis, can determine if an oocyst is sporulated or not. Oocysts are not infectious until they sporulate. The goal of this study was to design an automated model using Artificial Intelligence (AI) to expedite the process of enumeration, improve the efficiency and accuracy of the species identification, and determine the ability of the oocysts to infect. To this end, we trained and evaluated computer vision models based on the Mask RCNN neural network architecture. A model was trained to detect and differentiate three species and to determine sporulation for each (totaling six detection groups). This model achieved a mean relative percentage difference (RPD) of 5.64%, representing a slight overcount compared to manual counts, averaging across all groups. The mean RPD for each group individually fell within a range from −33.37% to 52.72%. These results demonstrate that these models were speedy and had high agreement with manual counts, with minimal processing of field-quality samples. These models also could differentiate the sporulation status of the oocysts, providing critical diagnostic information for potential field applications.

## 1. Introduction

Coccidiosis, a disease caused by *Eimeria* sp. parasites, is one of the most common diseases faced by the poultry industry worldwide [1,2]. Coccidia parasites can cause decreased growth rates, diarrhea, or even death. In addition, *Eimeria* sp. harm gut health and enable other enteric conditions, including clostridial enteritis. It is estimated that the annual economic impact of coccidiosis, including prevention, treatment, and production loss, is almost USD 2 billion in the United States alone, and over USD 15.5 billion worldwide [3].

The basis for successful control measures against coccidiosis relies on identification of these protozoal parasites. Typical methods for identifying and determining the severity of the protozoal infection include intestinal lesion scoring or enumeration of the protozoal oocysts in fecal samples [4]. While many poultry producers give little consideration to the species of coccidia, knowledge of the species can be important for developing vaccines or improving management strategies [5,6]. Standard methods to manually identify and enumerate these protozoal parasites require highly skilled technicians or veterinarians. This process is labor intensive, time-consuming, and susceptible to human error.

Automatic and semi-automatic protocols, including molecular tests, cytometry, and digital image analysis, for the enumeration and identification of coccidia are available [7,8,9,10]. Most PCR reactions target the intergenic transcribed spacer region 1 (ITS1) of the ribosomal RNA (rRNA) gene operon [7], a multi-copy gene, which makes these protocols unsuitable for estimation of the relative abundance of species in mixed infections of *Eimeria* sp. Vrba et al. [11] validated a quantitative PCR (qPCR) protocol to quantify samples with mixed *Eimeria* populations. However, to improve the sensitivity, the oocysts need to be sporulated beforehand, which can generate a delay in the results [12]. On the other hand, while there is no delay for protocols based on cytometry and digital image analysis, sample preparation can be cumbersome. Furthermore, if the samples contain debris of similar size to coccidia parasites, the number of oocysts can be overestimated. While improved preparation protocols may help to minimize these errors, the accuracy of the machine to differentiate between oocysts and debris cannot improve over time.

Poultry management and diagnostics is undergoing a significant transition with the introduction of machine learning or Artificial Intelligence models. These models have the potential to be an excellent diagnostic tool for coccidia identification, by exploiting *Eimerian* oocyst morphology. The human process of scanning for oocysts shapes and making decisions based on observation of morphological features (size, shape, and internal and external features) is simultaneous. This process is best modeled using a deep-learning-based approach, such as the Region-based Convolutional Neural Network-based (RCNN) model. This project aimed to demonstrate a computer vision model approach that would enable fast and accurate detection, speciation, and determination of sporulation status of coccidia oocysts without the need for extensive personnel training or subjectivity of traditional microscopic methods.

## 2. Materials and Methods

### 2.1. Coccidia Preparation

We prepared 30 samples of species-specific oocysts (*Eimeria acervulina*, *E. maxima*, and *E. tenella*, kindly donated by Dr. Mark Jenkins, USDA), for a total of 90 samples, by combining 100 μL of a pure isolate of coccidia species oocysts with 1 mL flotation solution (Feca-Med Sodium Nitrate, Vedco, Inc., St. Joseph, MO, USA). Additionally, we mixed 20 samples of sterile 100 μL of peptone buffer solution (Sigma-Aldrich, St. Louis, MO, USA) with 1 mL flotation solution.

To mimic field samples, we spiked 0.5 teaspoon (2.5 g approximately) fecal samples from poultry free of coccidia with 0.05 mL of a commercial coccidia vaccine (i.e., Advent^®^ by Huvepharma^®^, Peachtree City, GA, USA; Coccivac^®^-B52 by Merck & Co, Inc., Rahway, NJ, USA; Hatchpak^®^ Cocci III by Boehringer Ingelheim, Duluth, GA, USA; and Immucox^®^ 5 by Ceva Animal Health, Lenaxa, KS, USA). A minimum of 20 samples for each vaccine type will be prepared. The feces were then deposited in one side of a 7 oz (207 mL) whirl-pak filter bag (Nasco, Pleasant Prairie, WI, USA) that contained 22.5 mL Fecasol, to achieve a 1:10 dilution (feces to salt solution).

Each sample, including the negative mixture, was vortexed, centrifuged at 280 G, and allowed to sit for 10 min. Next, the samples were transferred from the top of each tube to a cell counter slide chamber (Countess™ Cell Counting Chambe Slide, Thermo Scientific, Waltham, MA, USA). We used an automated cell counter (Invitrogen Countess 3, Thermo Scientific, MA) to acquire images. The JPG photographs of the slides measured 2592 pixels (2257.63 μm) in width and 1944 pixels (1693.22 μm) length. We labeled the oocysts in the sample images using the LabelMe version 5.0.1 application (https://github.com/wkentaro/labelme, accessed on 10 August 2022). These manually labelled oocysts were used for training of the model. For each oocyst, labels indicated the *Eimeria* species and whether the oocyst was sporulated or not. Sporulated oocysts were identified by the presence of sporocysts within. On the other hand, non-sporulated oocysts were characterized by a single central mass (sporont) occupying most of the oocyst [13]. Manual enumeration, speciation, and sporulation status of all the samples were done by the same person. Counts were expressed as a total of units observed in each image, without transformations. Once labeling was complete, the LabelMe application saved the data as JPG and JSON file pairs, where each JSON file contained label information for its associated JPG file. These files formed the basis of the model training dataset.

### 2.2. Data Augmentation

To expand the training dataset, we performed multiple rotation and crop operations on all images and associated label data. First, we rotated each image at 45-degree intervals between 0 and 90 degrees (inclusive). Rotated images were cropped in-place to remove border artifacts from the rotation process. Next, we took multiple crops of each rotated image at fixed dimensions (1280 pixels horizontally, 960 pixels vertically). Consecutive crops used horizontal and vertical increments (425 pixel horizontal increment, 320 pixel vertical increment). These cropped images comprised the final dataset.

**Figure 1:**
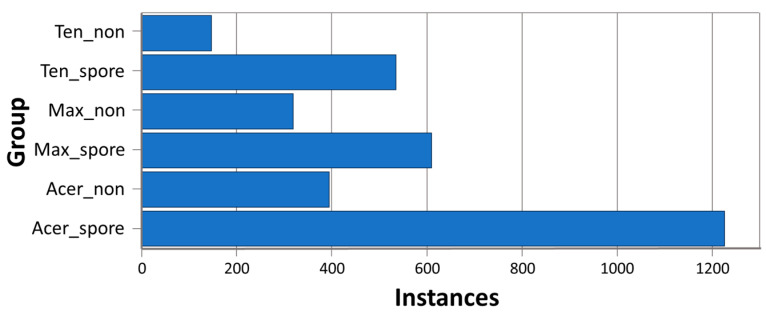
Instance counts for initial multi-species dataset. Oocyst counts for each group in the dataset used to train the multi-species models, across all images (*n* = 110), prior to any augmentation. Where Ten_non = *E. tenella* non-sporulated, Ten_spore = *E. tenella* sporulated, Max_non = *E. maxima*, non-sporulated, Max_spore = *E. maxima* sporulated, Acer_non = *E. acervulina* non-sporulated, and Acer_spore = *E. acervulina* sporulated.

To preserve ground-truth labels for training, the rotation and cropping parameters used on the images were reused to apply an equivalent transformation to the manual label data. This process enables a single large image with manual segmentation labels to be converted into a multi-image dataset made of many smaller training images with associated segmentation labels. To create the dataset used to train our multi-species models, we applied this process to an initial dataset of 110 images (Figure 1). This dataset consisted of 30 unchanged images of each *E. acervulina*, *E. maxima*, and *E. tenella* oocysts, for a total of 90 images. In addition, 20 images with no oocysts (negative) were included in the initial dataset. Each image was paired with its corresponding labeled file. Then we cropped and rotated the images, as described above, to expand the number of images to an augmented dataset of 2928 file pairs in the training dataset. Of this dataset, 732 file pairs were used in the validation dataset.

The dataset created using the process described here was further augmented during the training process by the Mask-RCNN library used for training.

### 2.3. Model Training and Performance

To train the model, we used PixelLib version 3.7 (https://github.com/ayoolaolafenwa/PixelLib accessed on 1 September 2022), a sophisticated open-source software library built for training Mask-RCNN models to perform instance segmentation. For the instance segmentation process, the software analyzed the group of pixels that made the labelled oocysts, rather than the individual pixels. As a starting point for training, we used Matterport’s Mask-RCNN 2.0 model (https://github.com/matterport/Mask_RCNN/releases/tag/v2.0 accessed on 1 September 2022) with MS COCO trained weights. For our implementation, we used the library’s default Learning Rate of 0.01, and adjusted the Learning Momentum to 0.95. For the models shown here, we used a batch size of 3 over 250 epochs. In other words, the system analyzed 3 images (batch size) concurrently before updating the internal model parameters. When it completes analyzing all the training images in the dataset, the model repeats the same process 250 times, also known as epochs. For each epoch, the system used updated model parameters based on the previous epoch. Our increased Learning Momentum (up from the library’s default value of 0.9) meant that the system considered the overall history of parameter changes to a greater degree for each subsequent epoch, making the system accelerate towards its final parameters more quickly. For feature extraction, we used ResNet101 as a network backbone.

To determine how similar the manually labelled and the model’s automatic counts were, we used mean relative percent difference (mRPD), according to the formula

$$mRPD=\frac{\sum_{i=1}^{n}\frac{a_{i}-m_{i}}{\frac{1}{2}\;\left(a_{i}+m_{i}\right)}}{n}\times 100\%$$

where *n* is the number of images in the dataset, *a_i_* is the AI model’s automatic count for the relevant group for image *i*, and *m_i_* is the ground-truth manual count for the relevant group for image *i*.

Note: To allow true-negative cases (where *a_i_* = *m_i_* = 0) to impact mRPD,

$$\frac{a_{i}-m_{i}}{\frac{1}{2}\;\left(a_{i}+m_{i}\right)}$$

was defined as 0 for these cases, corresponding to a mRPD of 0%. These cases indicate situations where the AI model correctly counted 0 instances of a group, and thus did not disagree with manual counts.

**Figure 2:**
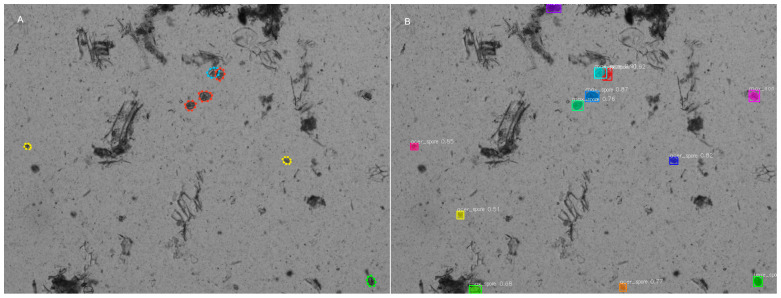
Comparison between an image of a multi-species sample that was manually labeled (**A**) and the automated output (**B**). Photo (**A**) is a manually labeled image used to train the model. For manual labels in (**A**), red is *E. maxima* sporulated, blue is *E. maxima* non-sporulated, yellow is *E. acervulina* sporulated), green is *E. tenella* sporulated. Image (**B**) shows the output from the multi-species model with confidence 0.5. Where label max_spore = *E. maxima* sporulated, max_non = *E. maxima* non-sporulated, acer_spore = *E. acervulina* sporulated, and tene_spore = *E. tenella* sporulated. The number adjacent to the label represents the model’s confidence of that classification (from 0 to 1, with 1 representing 100% certainty). The colors in this image are random to help differentiate individual oocysts, and do not relate to the species or sporulation status.

For an intuitive performance visualization, we also plotted ground-truth manual counts (X axis) against counts automatically generated from the model output (Y axis). Each point represents the counts for a specific group in each input image. Each chart has a regression line, with a reference line representing linear correlation (automatic count = manual count) at y = x. A regression line closer to y = x generally reflects better correlation between model counts and ground-truth counts for the input set. To measure the linear correlation of manual and automated oocyst counts, we used the Pearson correlation coefficient. We created Bland–Altman plots to visualize and quantify the agreement of the results between manual and AI-based methods. For correlation charts in Figure 2, and for mRPD calculations, we used the Pandas and Vega-Altair libraries for Python. We used JMP-Pro v.16 to calculate Pearson correlation coefficients and to create Bland–Altman plots.

## 3. Results

To evaluate the performance of the trained model we used mRPD, which assessed the relative difference between the counts from the manually labelled images and the model’s automatic counts (Table 1). Models trained on one species generally exhibited higher accuracy than models trained on multiple species. In a scale of 0–1, when the confidence threshold was 0.7, all the models for individual groups were over 90% accurate. In general, models recognized oocysts and ignored debris in images accurately (Figure 2). While there were a few false positive instances and misclassifications of oocysts, the model indicated a lower likelihood that these predictions were accurate by giving them a lower confidence score (below 0.7).

**Figure 3:**
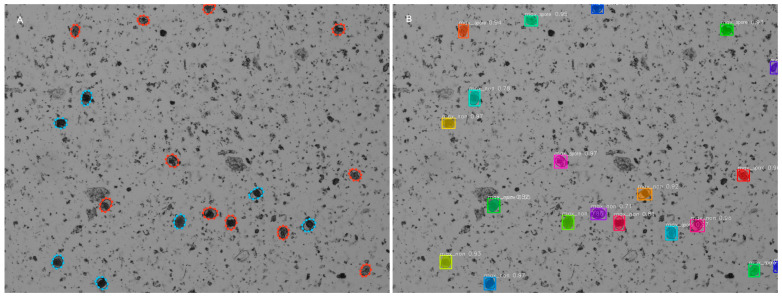
Comparison of manual labels and automated output for *E. maxima*. (**A**) Manually labelled oocysts, used to train the model. Red represents sporulated oocysts of *E. maxima*, blue indicates non-sporulated *E. maxima* oocysts. (**B**) Output from the multi-species model with confidence threshold set to 0.5. Where label max_spore = *E. maxima* sporulated and max_non = *E. maxima* non-sporulated. The number adjacent to the label represents the model’s confidence of that classification (from 0 to 1, with 1 representing 100% certainty). The colors in this image are random to help differentiate individual oocysts, and do not relate to the species or sporulation status.

Unsurprisingly, models produced the most accurate results when detecting only *E. maxima* oocysts. The multi-species model, when set to a confidence threshold of 0.5, correctly identified all the instances marked in our sample ground-truth reference for *E. maxima* (Figure 3). In a few instances, in samples that only had *E. maxima*, there was a mismatch between manual and automated identification. These mismatches generally occurred when the oocyst laid on the border of the image or two oocysts overlapped almost entirely. In the latter case, the automated system did not identify one of the instances. Very few of the visibly labeled instances were misclassified for sporulation.

**Figure 4:**
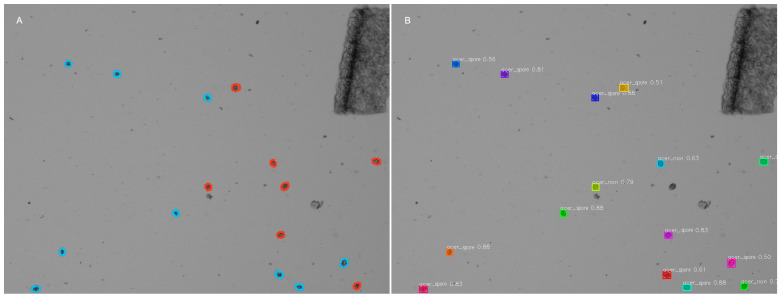
Comparison of manual labels and automated output for *E. acervulina*. Image (**A**) shows manually labeled oocyst, used to train the model. Blue represents *E. acervulina* sporulated oocysts, red indicates *E. acervulina* non-sporulated oocysts. Image (**B**) shows output from the multi-species model with confidence threshold set to 0.5. Where label acer_spore = *E. acervulina* sporulated, acer_non = *E. acervulina* non-sporulated oocysts. The number adjacent to the label represents the model’s confidence of that classification (from 0 to 1, with 1 representing 100% certainty). The colors in this image are random to help differentiate individual oocysts, and do not relate to the species or sporulation status. Here, the model manages to correctly identify 3 non-sporulated instances. Most other instances were misclassified as sporulated, though one non-sporulated instance remains unidentified.

On the other hand, when set to a confidence level of 0.5, the same multi-species model revealed discrepancies when identifying *E. acervulina* (Figure 4) and *E. tenella* oocysts. Most notably, this model over-identified sporulated *E. acervulina* oocysts, and under-identified non-sporulated *E. tenella* instances. Despite these tendencies, reducing the confidence threshold for the multi-species model from 0.7 to 0.5 resulted in a measurable improvement in overall RPD metrics. Considering all groups, the multi-species model with a confidence threshold of 0.5 had mean RPD values that were generally closer to 0, meaning that the manual and automatic counts are more similar.

**Figure 5:**
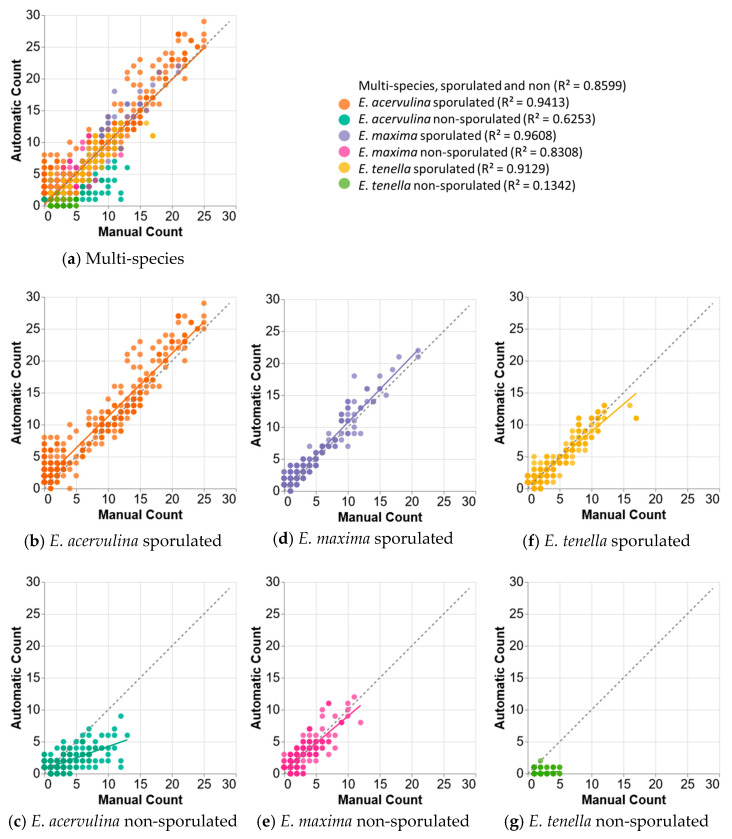
Pearson correlation between manual and automatic counts. Charts: Multi-species model (**a**) and all individual groups (**b**–**g**), at 0.5 confidence. For these charts, the multi-species model analyzed an input set that contained instances of all 3 *Eimeria* species, with both sporulated and non-sporulated instances of each species. The combined chart and most single group charts show good linear correlation. Chart (**a**) shows all groups in one chart, with groups separated by color. The number in parenthesis next to each group in the legend represents the linear regression R^2^ for sporulated and non-sporulated individual oocyst species. Counts are expressed as a total of units observed in each image.

Visualizations in Figure 5 show per-group correlation between manual and automated counting of oocysts for the most accurate multi-species model. The sporulated *E. acervulina* (acer_spore) group dominates the upper region of the chart (Figure 5b). This suggests that the models over-counted this group. On the other hand, the non-sporulated *E. acervulina* (acer_non) and the non-sporulated *E. tenella* (ten_non) groups dominate the lower region of this chart (Figure 5c,g), which suggests that the model under-counted these groups. Furthermore, the undercounts were worse as manual counts increased along the x-axis. Notably, the correlations between the manual and automated counts, for both the sporulated and non-sporulated, *E. maxima* (Figure 5d,e), as well as the sporulated *E. tenella* (tene_spore) (Figure 5f), were better and did not show any obvious trend towards under- or over-counting for these groups.

**Figure 6:**
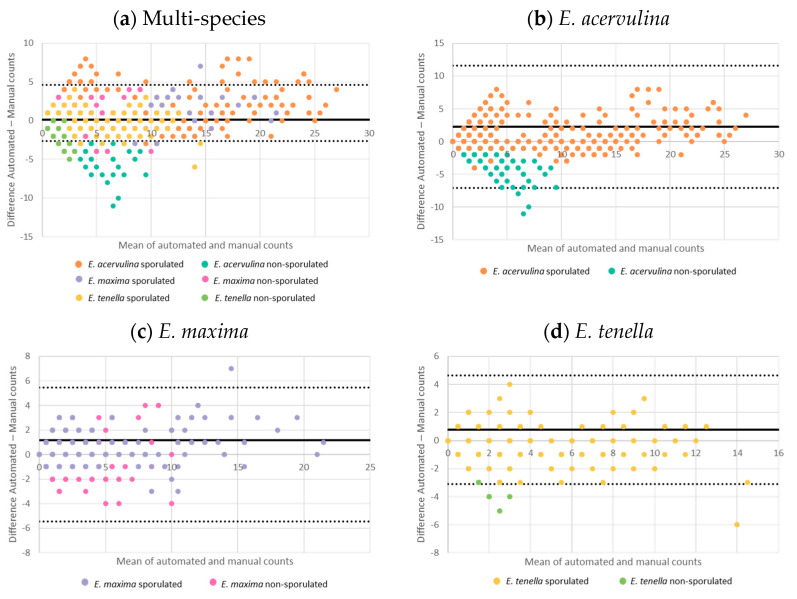
Bland–Altman plot comparing oocysts counts done automatically and manually: difference between both counts (Y axis) plotted against the mean difference between the counts (X axis). Counts were expressed as a total of units observed in each image, without transformations.

The Bland–Altman plots (Figure 6) show the agreement between manual and automatic counts. The solid line indicates the average difference between both counts. Under ideal conditions, the average between both counting methods is expected to be =0; dotted lines are the upper and lower 95% confidence interval for the mean. The closer the data points are to the mean line or are within the 95% confidence interval for the mean represent the agreement between the automated and manual (or ground-truth) counts.

Our model showed a high agreement (>95%) between the automated and manual counts of individual species. For *E. acerulina* oocysts, the agreement was 99.5% (or 729/732 samples within the 95% confidence internal), 99.79% (or 731/732 samples within the 95% confidence internal) for *E. maxima*, and 96.18% (or 728/732 samples within the 95% confidence internal) for *E. tenella*. For the *Eimeria* multi-species plot, the agreement was moderate (50–94%) between automated and manual counts, at 92.32% (or 128/1667 samples outside the 95% confidence internal). Furthermore, while each species showed high agreement, regardless of the sporulation status, the model showed poor agreement for the non-sporulated *E. acervulina* and *E. tenella*. This lack of agreement is in accordance with the lower correlation shown for these two groups.

## 4. Discussion

The purpose of this study was to compare manual and automated analyses, using a custom Mask-RCNN model, for the enumeration, speciation, and determination of sporulation status of three species of coccidia that infect chickens. We used digitalized microscopic images of floatation samples as a validation technique.

Enumeration and speciation of coccidia oocysts are commonly performed in research and even in some clinical investigations. Enumeration and speciation can be laborious because they are routinely performed manually. Consequently, this problem represents a substantial bottleneck for research projects and vaccine evaluations, requiring well-trained and experienced parasitologists. Validated qPCR protocols to quantify samples with mixed *Eimeria* populations are available [11]. However, the practical utility of PCR assays for routine diagnostics is questionable, because many PCR reactions described in the literature target the multi-copy ITS1 gene [7], which makes these protocols unsuitable for the quantification of oocysts in mixed infections. Results for protocols capable of quantifying samples with mixed *Eimeria* populations may be delayed because sporulation is recommended prior to performing the assay [11].

To date, there are a few automated protocols for oocyst enumeration and speciation using flow cytometry [9] and digital image analysis [10]. However, we found that the protocols used for sample preparation for these applications are complex, and the oocyst counts may be overestimated. A recent publication describes a fast and automated method for the enumeration of *Eimeria* oocysts [8]. None of the available protocols differentiates sporulated and non-sporulated oocysts. Information about the sporulation status can be important for clinicians and vaccine producers, as only sporulated oocysts can cause infection in poultry [13].

Recent advances in computer technology have enabled software-based automation of standard laboratory data analysis, including analysis of digital images. Artificial Intelligence represents a promising technology for microscopic parasite examination [14]. The Convolutional Neural Network (CNN) is the most commonly used artificial neural network to examine visual images [15]. Initial versions of our pipeline used OpenCV version 4.6 and TensorFlow CNN version 2.13 as foundational software packages to construct a complete working pipeline prototype. These packages were primarily used for image classification, object detection, and image segmentation tasks. We were able to prepare coccidia data by first labeling it, then using OpenCV to augment and expand it. Then we developed custom-trained models using TensorFlow. These models provided enumeration and speciation information for new input images in a process (inference) that again used OpenCV.

Once we had the prototype, we used the Mask-RCNN pipeline for the image analysis. Mask-RCNN is a newer CNN algorithm that makes object detection and its classification more accurate and faster [16]. Our machine learning pipeline for the identification and enumeration of coccidia parasites constituted three steps: data preparation, training, and inference. As with any machine learning pipeline, dataset preparation was the key to successfully achieving a high-accuracy model [17]. When creating such datasets, labeling images is often time-consuming. In this study, data labeling means to identify the species of each oocyst and whether it is sporulated or not. For the model to extract the proper information, accurate labeling is essential. For example, the model must learn to discriminate debris from oocysts, determine the species of *Eimeria*, or whether the oocyst is sporulated or not. Also, we needed to label as many oocysts as possible, including partial ones at the margin of the slide and overlapping oocysts, to prevent the algorithm from misclassifying them. Irrelevant features, such as the orientation of the oocysts, can also interfere with data extraction during training, and affect the results. Data enhancement, including horizontal flipping, aims to teach the model to learn the invariance of the data [18]. A weakness of our model was the inability to include images with no oocysts, as PixelLib, our training library, rejected these images. This difficulty should be further investigated, and solutions further developed.

Training the application by species was the most time intensive. This required a thoughtful approach to (1) obtaining examples of manually labeled oocyst and (2) devising a protocol that would provide adequate data to the learning algorithms without becoming overly burdensome for humans. In other words, our efforts focused on making each manually labeled image as useful as possible in building a generalizable vision model that would perform accurately and reliably on novel samples. For this step, we used 80% of file pairs from the augmented dataset. The other 20% of the file pairs were used for validation and fine tuning of the model. We used novel ways of manipulating the labeled images to improve the accuracy of the computer vision model without needing a large number of labeled images. The original dataset contained 110 file pairs that, after manipulation, were expanded to 2928 file pairs.

The Learning Rate and Learning Momentum parameters are important in identifying over-learning and when to stop training [19]. In our model, a small Learning Rate of 0.01 and a Learning Momentum of 0.95 provided the appropriate combination for the performance prediction. The batch size determines the training time, memory usage, and accuracy. There is no single “best” batch size. A small batch size may be slower to train but consumes less memory and provides more accurate results. For this reason, we selected a batch size of three images. For the number of epochs, we started with 18 epochs, and we increased the number until the model no longer improved.

Finally, we tested our model with samples prepared from three commercial coccidia vaccines that contained the three *Eimeria* spp. Our approach was consistently able to achieve good performance for single species samples. The automated oocyst counts showed high agreement with the counts obtained by the manual method for the individual species models. On the other hand, for the multi-species model, the automated oocyst counts showed moderate agreement compared to the manual method. Between all models for each of the three species, the average deviation from the correct count ranged from an average underestimate of 0.18% to an average overestimate of 0.39%. The poorest agreement was in the non-sporulated *E. acervulina* and *E. tenella* oocysts. This is not surprising, as these oocysts are smaller than those of *E. maxima*, and it may be difficult to identify the sporocysts within the oocysts. Further, our training dataset contained fewer instances of these groups compared to some other groups.

We wanted our model to be capable of differentiating sporulated and non-sporulated oocysts, because only the first are infective. This sporulation step occurs in the environment. While sporulated oocysts may survive in the environment for more than a year, non-sporulated oocysts can only survive a short time in the environment [20]. If environmental conditions prevent the oocysts from sporulating, it is possible that a vaccinated flock does not get good coverage and a coccidia outbreak occurs later in life, when losses can be more severe. An automated model for speciation of *Eimeria* sp. oocysts, with an accuracy of 96.9%, has been published recently [21]. For this validation model, these researchers used a publicly available database (http://www.coccidia.icb.usp.br/ accessed on 15 January 2023 [22], which only contains sporulated oocysts. Therefore, while this model may identify coccidia by species, it cannot differentiate sporulation status of the oocysts, which can be important for assessing the management conditions, vaccine cycling, or quality of a vaccine.

To differentiate between sporulated and non-sporulated coccidia, we fine-tuned the parameters of the neural network incorporating the sporulation status as a new group, thus resulting in six different group-labels (one for each of the three species in one of two sporulation states). We experimented by creating single-species models that could differentiate sporulated from non-sporulated oocysts, as well as creating a combined model capable of both speciating and determining sporulation simultaneously.

The single-species and multi-species models excelled in identifying *E. maxima* oocysts. Furthermore, the models were also successful in differentiating sporulated and non-sporulated oocysts of *E. maxima*. This is expected as these species are the largest [13] and easy to resolve visually and by digital computing. On the other hand, the model was least accurate for *E. acervulina*, which has the smallest oocysts of the three species investigated. Notably, most of the false positives had a low confidence value (below 0.7). A higher confidence threshold might have produced more accurate speciation results by eliminating these false positive detections. Furthermore, to develop a successful model, it is most important to have digital images that have high resolution, that is in focus. Therefore, better imaging solutions that produce higher-resolution images are another potential avenue for improvement.

Important components of successful analytical methods used in routine diagnosis and research include performance, time, and resource-efficiency. Our results show that automated image analysis is promising, and it can drastically reduce the analysis time compared to manual measurements. The model demonstrated good accuracy, with just a few digital images presented. Further validation of the Artificial Intelligence model would enable accurate and rapid analysis of a larger number of samples in a short period of time. This would decrease the risk of bias by the analyst.

This study was a proof of concept to demonstrate we can classify and enumerate coccidia using AI. We worked with the three most common coccidia, which are included in all the commercial vaccines and whose oocyst morphology is relatively easy to separate visually. In the future, we plan to include other *Eimeria* spp. in the model. It would be interesting to develop a model that is capable of discriminating oocysts from *Eimeria* protozoa that have similar dimensions and sizes, such as *E. tenella* vs. *E. brunetti*, and *E. acervulina* vs. *E. praecox*. Similarly, we would like to develop a model for turkey coccidia, whose oocysts are difficult to separate visually and biochemically [23].

## 5. Conclusions

In conclusion, the proposed AI model enables rapid and automated enumeration, speciation, and identification of sporulation of three species of coccidia oocysts of chickens from light-microscopic images with overall excellent precision and accuracy for *E. maxima* oocysts, as well as sporulated oocysts of *E. acervulina* and *E. tenella*. However, the model was weak identifying non-sporulated *E. acervulina* and *E. tenella*. Two factors that might have contributed to the poor correlation of the model are the image resolution due to the small size of the oocyst as well as the low numbers of non-sporulated oocysts in the samples used for training and inference. Higher-resolution images may be needed to improve the model. Currently, additional samples are being analyzed to improve the accuracy of the model. Another shortcoming of our model was the inability to analyze samples with no oocysts. The cause of rejection of these samples is being investigated and solutions will be developed. Even with these weaknesses, this method, based on open-source software, has the potential to significantly reduce the time needed for the enumeration and speciation of coccidia as well as to help with coccidia research and development of vaccines.

Although still in its infancy, AI image analysis is likely to be a key technology in the future. In the long term, this technology can lead to the development of other industry assays, such as more accurate identification of shedding patterns of specific species of coccidia in mixed infections, and evaluation of vaccines and anticoccidial drugs. This system could be expanded to identify other parasites and even be used in the diagnosis of intestinal parasites in other animal species.

## Figures and Tables

**Table 1:** Relative Percent Difference (RPD) between the manual and the model’s automatic counts. This value was used to assess the performance for single- and multi-species models. Each model was trained on a dataset containing oocyst examples of each group listed for that model. The model counted as oocysts the units that were above the set confidence threshold. Mean RPD values closer to 0 indicate better consensus between both manual and automatic counts.

Model Type	Model Confidence Threshold	No. of Images in Input Dataset	Group ^1^	Mean % RPD
Single-Species				
*E. acervulina*	0.7	140	Acer_spore	1.26
			Acer_non	−7.01
*E. maxima*	0.7	141	Max_spore	4.49
			Max_non	8.17
*E. tenella*	0.7	129	Ten_spore	4.03
			Ten_non	−6.20
Multi-Species (*E. acervulina*, *E. maxima*, *E. tenella*)	0.7	732	Acer_spore	25.32
			Acer_non	−47.40
			Max_spore	−1.18
			Max_non	−9.21
			Ten_spore	−10.89
			Ten_non	−37.61
	0.5	732	Acer_spore	52.72
			Acer_non	−4.40
			Max_spore	7.16
			Max_non	1.58
			Ten_spore	10.13
			Ten_non	−33.37

^1^ Acer_spore = *E. acervulina* sporulated, Acer_non = *E. acervulina* non-sporulated Max_spore = *E. maxima* sporulated, Max_non = *E. maxima* non-sporulated, Ten_spore = *E. tenella* sporulated, and Ten_non = *E. tenella* non-sporulated.

## Data Availability

The data presented in this study are available on request from the corresponding author. The data are not publicly available due to [patent application].

## References

[1] Fatoba A.J., Adeleke M.A. (2018). Diagnosis and control of chicken coccidiosis: A recent update. J. Parasit. Dis..

[2] Jones P.J., Niemi J., Christensen J.-P., Tranter R.B., Bennett R.M. (2019). A review of the financial impact of production diseases in poultry production systems. Anim. Prod. Sci..

[3] Blake D.P., Knox J., Dehaeck B., Huntington B., Rathinam T., Ravipati V., Ayoade S., Gilbert W., Adebambo A.O., Jatau I.D. (2020). Re-calculating the cost of coccidiosis in chickens. Vet. Res..

[4] Conway D.P., McKenzie M.E. (2007). Poultry Coccidiosis Diagnostic and Testing Procedures.

[5] McDougald L.R., Fuller L., Solis J. (1986). Drug-sensitivity of 99 isolates of coccidia from broiler farms. Avian Dis..

[6] Lee B.H., Kim W.H., Jeong J., Yoo J., Kwon Y.K., Jung B.Y., Kwon J.H., Lillehoj H.S., Min W. (2010). Prevalence and cross-immunity of *Eimeria* species on Korean chicken farms. J. Vet. Med. Sci..

[7] Jenkins M.C., Miska K., Klopp S. (2006). Application of polymerase chain reaction based on ITS1 rDNA to speciate *Eimeria*. Avian Dis..

[8] Smith M.K., Buhr D.L., Dhlakama T.A., Dupraw D., Fitz-Coy S., Francisco A., Ganesan A., Hubbard S.A., Nederlof A., Newman L.J. (2023). Automated enumeration of *Eimeria* oocysts in feces for rapid coccidiosis monitoring. Poult. Sci..

[9] Adams D.S., Kulkarni R.R., Mohammed J.P., Crespo R. (2022). A flow cytometric method for enumeration and speciation of coccidia affecting broiler chickens. Vet. Parasit..

[10] Adams D.S., Ruiz-Jimenez F., Fletcher O.J., Gall S., Crespo R. (2022). Image analysis for *Eimeria* oocyst counts and classification. J. Appl. Poult. Res..

[11] Vrba V., Blake D.P., Poplstein M. (2010). Quantitative real-time PCR assays for detection and quantification of all seven *Eimeria* species that infect the chicken. Vet. Parasit..

[12] Edgar S.A. (1955). Sporulation of oocysts at specific temperatures and notes on the prepatent period of several species of avian coccidia. J. Parasitol..

[13] Cervantes H.M., McDougald L.R., Jenkins M.C., Swayne D.E., Boulianne M., Logue C.M., McDougald L.R., Nair V., Suarez D.L. (2020). Coccidiosis. Diseases of Poultry.

[14] Lebel P., Dial R., Vemuri V.N.P., Garcia V., DeRisi J., Gómez-Sjöberg R. (2021). Label-free imaging and classification of live *P. falciparum* enables high performance parasitemia quantification without fixation or staining. PLoS Comput. Biol..

[15] Bharati P., Pramanik A. Deep Learning Techniques—R-CNN to Mask R-CNN: A Survey. Proceedings of the Advances in Intelligent Systems and Computing.

[16] He K., Gkioxari G., Dollár P., Girshick R. Mask R-CNN. Proceedings of the IEEE International Conference on Computer Vision (ICCV).

[17] Krell M.M., Seeland A., Kim S.-K. Rotational data augmentation for electroencephalographic data. Proceedings of the 39th Annual International Conference of the IEEE Engineering in Medicine and Biology Society (EMBC).

[18] Cubuk E.D., Zoph B., Mane D., Vasudevan V., Le Q.V. AutoAugment: Learning augmentation policies from data. Proceedings of the IEEE/CVF Conference on Computer Vision and Pattern Recognition (CVPR).

[19] Attoh-Okine N.O. (1999). Analysis of learning rate and momentum term in backpropagation neural network algorithm trained to predict pavement performance. Adv. Eng. Softw..

[20] Price K.R., Guerin M.T., Barta J.R. (2014). Success and failure: The role of relative humidity levels and environmental management in live *Eimeria* vaccination of cage-reared replacement layer pullets. J. Appl. Poult. Res..

[21] He P., Chen Z., He Y., Chen J., Hayat K., Pan J., Lin H. (2023). A reliable and low-cost deep learning model integrating convolutional neural network and transformer structure for fine-grained classification of chicken *Eimeria* species. Poult. Sci..

[22] Castañón C.A.B., Fraga J.S., Fernandez S., Gruber A., Costa L.d.F. (2007). Biological shape characterization for automatic image recognition and diagnosis of protozoan parasites of the genus *Eimeria*. Pattern Recognit..

[23] Gadde U.D., Rathinam T., Finklin M.N., Chapman H.D. (2019). Pathology caused by three species of *Eimeria* that infect the turkey with a description of a scoring system for intestinal lesions. Avian Pathol..

